# Recurrence Quantitative Analysis of Wavelet-Based Surrogate Data for Nonlinearity Testing in Heart Rate Variability

**DOI:** 10.3389/fphys.2022.807250

**Published:** 2022-02-09

**Authors:** Martín Calderón-Juárez, Gertrudis Hortensia González Gómez, Juan C. Echeverría, Héctor Pérez-Grovas, Eduardo Quintanar, Claudia Lerma

**Affiliations:** ^1^Plan de Estudios Combinados en Medicina, Facultad de Medicina, Universidad Nacional Autónoma de México, Mexico City, Mexico; ^2^Departamento de Instrumentación Electromecánica, Instituto Nacional de Cardiología Ignacio Chávez, Mexico City, Mexico; ^3^Departamento de Física, Facultad de Ciencias, Universidad Nacional Autónoma de México, Mexico City, Mexico; ^4^Departamento de Ingeniería Eléctrica, Universidad Autónoma Metropolitana, Unidad Iztapalapa, Mexico City, Mexico; ^5^Departamento de Nefrología, Instituto Nacional de Cardiología Ignacio Chávez, Mexico City, Mexico

**Keywords:** recurrence analysis, surrogate data, nonlinear dynamics, nonstationarity, heart rate variability, hemodialysis, active standing

## Abstract

Exploring the presence of nonlinearity through surrogate data testing provides insights into the nature of physical and biological systems like those obtained from heart rate variability (HRV). Short-term HRV time series are of great clinical interest to study autonomic impairments manifested in chronic diseases such as the end stage renal disease (ESRD) and the response of patients to treatment with hemodialysis (HD). In contrast to Iterative Amplitude Adjusted Fourier Transform (IAAFT), the Pinned Wavelet Iterative Amplitude Adjusted Fourier Transform (PWIAAFT) surrogates preserve nonstationary behavior in time series, a common characteristic of HRV. We aimed to test synthetic data and HRV time series for the existence of nonlinearity. Recurrence Quantitative Analysis (RQA) indices were used as discriminative statistics in IAAFT and PWIAAFT surrogates of linear stationary and nonstationary processes. HRV time series of healthy subjects and 29 ESRD patients before and after HD were tested in this setting during an active standing test. Contrary to PWIAAFT, linear nonstationary time series may be erroneously regarded as nonlinear according to the IAAFT surrogates. Here, a lower proportion of HRV time series was classified as nonlinear with PWIAAFT, compared to IAAFT, confirming that the nonstationarity condition influences the testing of nonlinear behavior in HRV. A contribution of nonlinearity was found in the HRV data of healthy individuals. A lower proportion of nonlinear time series was also found in ESRD patients, but statistical significance was not found. Although this proportion tends to be lower in ESRD patients, as much as 60% of time series proved to be nonlinear in healthy subjects. Given the important contribution of nonlinearity in HRV data, a nonlinear point of view is required to achieve a broader understanding of cardiovascular physiology.

## Introduction

Exploring the presence of nonlinearity in data provides insights into the nature of physical and biological systems (Schreiber and Schmitz, 2000). With this aim, surrogate data testing as proposed by Theiler et al. (1992), is applied by the creation of several versions of time series that no longer involve nonlinearity despite preserving statistical properties. A nonlinear statistical measure is then applied to the surrogates and the original time series; any deviation of this measure in the surrogates as compared to the one obtained from such original series is used to discriminate this series from the null hypothesis, which is met by the linear surrogates. Other authors have also proposed to improve the null hypothesis to address more specific types of behavior (Lancaster et al., 2018). Fourier transform-based surrogates generate constrained realizations that virtually preserve the same correlation function of the original data. The statistical null hypothesis of the Iterative Amplitude Adjusted Fourier Transform (IAAFT) technique is that the data represent a stationary linear Gaussian process, measured through an invertible, time-independent instantaneous measurement function (Schreiber and Schmitz, 1996; Lancaster et al., 2018). This technique has been extensively used in physical and biological systems, such as in the analysis of heart rate variability (HRV) (Porta et al., 2015; Faes et al., 2019).

HRV refers to the instantaneous changes in heart rate, measured as the time interval of consecutive R waves in electrocardiography (ECG) recordings, and it is a powerful and simple tool for the study of cardiovascular physiology (No authors listed, 1996). HRV is used in clinical studies and, through decades, it has been considered in various medicine applications (Sassi et al., 2015). Short-term HRV recordings are analyzed for the study of the autonomic nervous system and its influence on the cardiovascular system (No authors listed, 1996; Sassi et al., 2015). The approach of IAAFT surrogates has been used in short-term HRV time series (Porta et al., 2015; Faes et al., 2019), as well as other Fourier transform-based surrogates (Yamamoto et al., 1993; Porta et al., 2000; Lerma et al., 2003; Faes et al., 2004), both offering different results regarding the presence of nonlinear behavior in HRV data.

One of the main disadvantages of Fourier transform-based surrogates is that the original time series must be limited to stationary processes (Borgnat and Flandrin, 2009), while HRV is often nonstationary (Porta et al., 2004). Some other approaches for surrogate data testing consider, as well, the condition of nonstationarity in their null hypothesis (Keylock, 2006; Faes et al., 2009; Lucio et al., 2012). One of them is Pinned Wavelet Iterative Amplitude Adjusted Fourier Transform (PWIAAFT) (Keylock, 2007), which conserves the nonstationary behavior in the surrogate data in a controlled fashion. The analysis of nonlinearity in these settings has led to the increasing application of nonlinear tools in which time series are not required be neither very long nor nonstationary; this is the case of the recurrence plots (RP) (Marwan et al., 2002, 2007). Recurrence quantitative analysis (RQA) is used to quantify diverse nonlinear behaviors in the RP and is widely used in physiological time series, such as electroencephalography (EEG) (Ouyang et al., 2008; Heunis et al., 2018; Pitsik et al., 2020) and ECG (Marwan et al., 2002; Naschitz et al., 2003; Gonzalez et al., 2013; Martín-González et al., 2018).

To study the influence of the autonomic nervous system on cardiovascular dynamics by HRV analysis, the active standing test is used for eliciting controlled parasympathetic predominance at supine position and a sympathetic influence during active standing (Carnethon et al., 2002). Also, it has been of great clinical interest to study autonomic impairments manifested in chronic diseases such as the end stage renal disease (ESRD) (Echeverria et al., 2017; Bokhari et al., 2018). ESRD patients treated with hemodialysis (HD) are in fact subjected to a significant physiological stress during each HD session (Kooman et al., 2018), which involves a sympathetic “challenge” on a regular basis (Lerma et al., 2015) and thus becomes a robust model for the study of autonomic impairments. According to changes indicated by RQA indices during an active standing test, the cardiovascular dynamics associated with both ESRD and HD are consistent with the loss of access to some dynamic physiological conditions (Gonzalez et al., 2013; Calderon-Juarez et al., 2020). Furthermore, recent reports of the correlation between the mean duration of the cardiac cycle (meanNN) with RQA indices in HRV time series (Calderon-Juarez et al., 2020; Robles-Cabrera et al., 2021), suggest that the meanNN parameter, which reflects changes in the cardiac activity required to address different hemodynamic challenges, may influence the nonlinear dynamics of HRV as well. Yet, it has not been fully demonstrated whether the RQA indices in short-term HRV time series exhibit nonlinear dynamics by using surrogate data approach, in particular, considering the nonstationary behavior of these time series.

The purpose of this work was to assess RQA indices as discriminative nonlinear statistics using IAAFT and PWIAAFT surrogates applied to short-term HRV time series of healthy subjects and ESRD patients (before and after treatment with HD) collected during an active standing test.

## Materials and Methods

### Synthetic Data

Given that, *a priori*, the type of dynamical behavior of HRV time series was unknown, linear synthetic signals were first tested to ensure that a proper confirmation of the null hypothesis in stationary and nonstationary settings was achieved by the combination of RQA and the algorithms used for the generation of surrogate data; thereby preventing misinterpretations of false detections of nonlinearity in HRV data. The following second order autoregressive (AR2) processes were used as controls for surrogate data testing. These are the same used by Keylock (2007): (a) AR2s, AR2 process with broad energy spectrum, considered as stationary Eq. (1); (b) AR2ns, AR2 process with a peaked energy spectrum Eq. (2). Using these processes, one thousand and eight hundred (1800) values were obtained and the first 1500 values were discarded from both AR2 processes, avoiding transient changes at the beginning of the time series. The remaining 300 values were thus considered to evaluate the linear null hypothesis in synthetic time series, as those of short segments of HRV data, in which near 300 heartbeat intervals are typically contained.


(1) $$x\,\left(t\right)=0.8\,x_{t-1}-0.25\,x_{t-2}+\varepsilon$$

(2) $$x\,\left(t\right)=1.59\,x_{t-1}-0.96\,x_{t-2}+\varepsilon$$

### Heart Rate Variability Time Series

#### Study Protocol

Electrocardiography (ECG) recordings were obtained following the protocol described by Calderon-Juarez et al. (2020). These recordings were obtained during an active orthostatic test from healthy subjects and ESRD patients, as described below. Continuous one-channel ECG recordings were collected during 10 min in supine position followed by subsequent recordings during further 10 min of active standing. The final 5 min of each recording were selected as representative data segments of supine position and active standing, respectively. Patients maintained spontaneous breathing during all procedures. ECG recordings were obtained at 250 samples per second and the identification of R waves was achieved by a second derivative algorithm. The periods of consecutive heart cycles are commonly known as NN or RR intervals, which in turn form the HRV time series. Finally, a correction of artifacts was visually supervised in these series and any outliers by the existence of ectopic beats were replaced using linearly interpolated intervals.

#### Participants

Forty recordings were obtained in healthy subjects, age 32 years (27–37, CI 95%), body mass index (BMI) 22.06 kg/m^2^ (20–24, CI 95%), proportion of males 34.5%. Twenty-nine ESRD patients were included, age 26 years (24–30, CI 95%, *p* = 0.084 vs. healthy), BMI 23.3 kg/m^2^ (22–25, CI 95%, *p* = 0.053 vs. healthy), proportion of males 51.2% (*p* = 0.295 vs. healthy group). End stage renal disease patients were studied before and after treatment with hemodialysis following the same active orthostatic test protocol. These patients were studied in a previous work (Calderon-Juarez et al., 2020). Hemodialysis sessions had a mean duration of 3.6 ± 0.5 h with total volume removal of 3.1 ± 1.1 L. HD vintage was 12.5 ± 10.2 months with a residual renal function of 0.9 ± 1.5 mL/min. Laboratory results within 1 month prior to the study (obtained from blood samples taken on any day when hemodialysis was not performed) showed creatinine = 8.7 ± 2.5 mg/dL, potassium = 4.9 ± 0.7 mEq/L, phosphorous = 5.1 ± 1.5 mEq/dL, calcium = 8.9 ± 1.1 mg/dL, hemoglobin = 8.3 ± 2.7 g/dL, albumin = 3.9 ± 0.5 g/dL, cholesterol = 165 ± 41 mg/dL, and triglycerides = 145 ± 96 mg/dL. The ESRD etiologies for these patients were systemic lupus erythematosus (*n* = 1), focal segmental glomerulosclerosis (*n* = 1), or unknown (*n* = 27). All procedures following the ethical standards of the 1964 Helsinki’s declaration in its later amendments. Our protocol was approved by the Research and Ethics Committee of the Instituto Nacional de Cardiología Ignacio Chávez (protocol number 21-1236). Informed consent was obtained from all participants.

#### Hemodialysis Prescription

Hemodialysis (HD) sessions were delivered with volumetric dialysis machines (4008 H, Fresenius Medical Care, Bad Homburg, Germany) using ultrapure dialysate (HCO${}_{3}^{-}$ = 35 mmol/L, Na^+^ = 138 mmol/L, K^+^ = 2 mmol/L, Ca^2 +^ = 3.5 mEq/L, Mg^2 +^ = 1.0 mEq/L) and polysulfone membranes (F-60 and F-80, Fresenius Medical Care, Walnut Creek, CA, United States). Hypertension was controlled by strict prescription of dry body weight without using antihypertensive drugs following an approach of extracellular volume control by convection. Patients were on a non-restrictive diet and did not use erythropoietin.

#### Heart Rate Variability Time and Frequency Domain Indices

HRV traditional indices for this study protocol have been reported previously (Gonzalez et al., 2013; Calderon-Juarez et al., 2020). To provide a broad characterization of HRV in the subjects and patients, time domain and frequency domain indices were also calculated here. The meanNN index is the mean value of all RR intervals contained in the time series and SDNN is the standard deviation. Power spectral indices were computed by the Fourier transform method, resampling at 3 Hz and applying a non-overlapped Hamming window of 300 data points with 50% overlap. The Low Frequency band (LF) corresponds to frequencies 0.04–0.15 Hz, and the High Frequency band (HF) corresponds to 0.15–0.4 Hz. High Frequency band is tightly related with parasympathetic activity, whereas LF corresponds to a combination of sympathetic and parasympathetic influence (No authors listed, 1996). We report the LF/HF ratio to express the autonomic modulation as a succinct expression (No authors listed, 1996).

### Recurrence Quantitative Analysis

The RQA is based on the construction of recurrence plots, defined by Marwan et al. (2007) :


(3) $$R_{i,j}=\Theta \left(\varepsilon_{i}-||\vec{x_{i}}-\vec{x_{j}}||\right),\vec{x_{i}}\in {\mathbb{R}}^{m},i,j=1,,N,$$

where N is the number of considered states *x*_*i*_, ε_*i*_ is a threshold distance, ||⋅|| a norm and Θ (⋅) is the Heaviside function.

As described thoroughly by Trauth et al. (2019), the representation of multidimensional systems from one-dimensional time series by the time delay embedding approach preserves the dynamic characteristics of the system (Packard et al., 1980). Given that the embedding dimension is sufficiently large, the reconstructed phase space does preserve the topological characteristics of the real phase space (Packard et al., 1980; Takens, 1981). The norm to establish the vicinity for the construction of the recurrence plot must be defined though. Probably, the most common one is the Euclidean norm (the neighborhood is a sphere), in which ε is the radius that contains a fixed number of states (Marwan et al., 2007; Trauth et al., 2019). To study the behavior of nonstationary signals, the fixed amount of nearest neighbors (FAN), in which the radius ε changes for each point, leads to an asymmetric recurrence plot in which all columns have the same recurrence density despite the nonstationary behavior, or trends, in the time series (Marwan, 2011). Therefore, to address this phenomenon, FAN norm has been recommended for analyzing HRV (Martín-González et al., 2018).

The embedding parameters of the time series in this work were calculated with the function of false nearest neighbors (embedding dimension – m) and correlation function (embedding delay – τ). The value of *m* and τ were selected at the point where the false nearest neighbors and the correlation function reached their first local minimum at zero, respectively (Calderon-Juarez et al., 2020). These parameters were calculated for each time series, and the same set of values were used for RQA of surrogate data. After applying the embedding method for reconstructing the attractor of each HRV time series into the phase space, RPs were obtained with an ε = 0.07, the FAN norm, a Theiler window = τ, window shift = 1 and minimal length of diagonal and vertical lines = 2.

We used the CRP toolbox for MATLAB provided by Marwan et al. (2007), available at (http://tocsy.agnld.uni-potsdam.de/crp.php). The following RQA indices were obtained: recurrence rate (RR), determinism (DET), averaged diagonal length (ADL), length of longest diagonal line (LLDL), entropy of diagonal length (ENT) (Marwan et al., 2007), laminarity (LAM), trapping time (TT) (Marwan and Kurths, 2002), length of longest vertical line (LLVL), recurrence time of the 1st type (T1), recurrence time of the 2nd type (T2) (Gao and Cai, 2000), recurrence period density entropy (RPDE), clustering coefficient (CC) (Marwan et al., 2009) and transitivity (TRANS) (Donner et al., 2010), see Appendix A for definition of RQA indices. For meanNN correlations with RQA indices in surrogate data, the mean values of the RQA indices from the 99 generated surrogates for every subject were obtained.

### Stationarity Testing

The existence of restricted weak stationarity (i.e., steady mean and variance) was tested in the synthetic data and original HRV time series to assess the potential implications for surrogate testing of analyzing data with a nonstationary behavior. We followed the algorithm proposed by Porta et al. (2004). A Kolmogorov-Smirnov test goodness-of-fit was used to evaluate if a normal distribution was present in time series, otherwise a logarithmic transformation was applied. N-L+1 ordered sequences of length L were used to create a randomly selected M number of segments or subsets. The length N was set to 300 data points in accordance with the above-mentioned autoregressive processes. L was set to 50 data points to observe at last 5 cycles of LF (about 0.1 Hz); eight M subsets were taken at random to increase the possibility of selecting subsets covering the full extent of time series. After this selection, for the time series with a normal distribution, the stability of the mean and variance was checked by analysis of variance (ANOVA) and Bartlett tests, respectively. For time series with no normal distribution, the stability of the mean and variance was tested using Kruskal-Wallis and Levene tests, respectively. Statistical differences for all tests were considered at the confidence level of *p* < 0.05.

### Surrogate Testing

The IAAFT described by Schreiber and Schmitz (1996) was used for the generation of stationary surrogates with MATLAB toolbox provided in Lancaster et al. (2018). PWIAAFT surrogates were generated with a threshold (ρ) of 0, 0.01, 0.03, and 0.3, which were the same explored in Keylock (2006, 2007). We followed the routine described in detail by Keylock et al. (2011) and used the MATLAB toolbox provided by this author available at (https://sites.google.com/site/chriskeylocknet/software/surrogate-generation-algorithms/pwiaaft). Ninety-nine surrogates were generated from each original time series, being either synthetic data [obtained from Eqs. (1) and (2)] or HRV data (obtained from participants), to achieve a two-sided α error of 0.01. Statistically significant differences of surrogate data testing were considered when the statistic of the original time series was *p* < 0.05.

### Statistical Analysis

Categorical variables are reported as percentages and were compared between healthy subjects and patients by exact Fischer’s tests. For the comparison among the study groups (healthy, ESRD before HD and ESRD after HD), positions (supine and active standing) and surrogate technique (IAAFT and PWIAAFT) a *post hoc* correction was done by the Bonferroni method. In other words, we compared the proportion of nonlinear time series in IAAFT vs. PWIAAFT (same group and position), supine position vs active standing, healthy vs. ESRD before HD (same position), healthy vs ESRD after HD (same position) and ESRD before HD vs. ESRD after HD (same position). For continuous variables, normal distribution was assessed through Kolmogorov-Smirnov test, median (95% confidence interval) are expressed and were compared with Mann-Whitney U test. Bivariate correlations were tested by the Spearman correlation coefficient. The statistical analyses were performed with the Statistical Package for the Social Sciences (SPSS) version 26, and p-values <0.05 were considered as significant.

## Results

### Synthetic Data

#### Stationarity Testing

**FIGURE 1:**
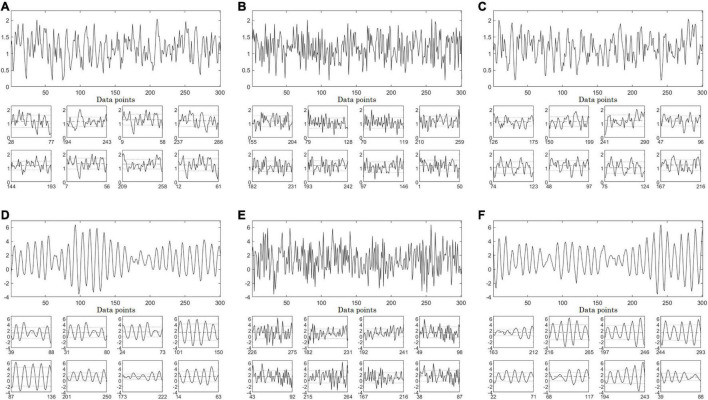
Full-size panels depict synthetic time series, original (left column), one Iterative Amplitude Adjusted Fourier Transform (IAAFT) surrogate time series (middle column) and one Pinned Wavelet Iterative Amplitude Adjusted Fourier Transform (PWIAAFT) (ρ = 0.01) surrogate time series (right column). AR2s Eq. (1) time series corresponds to top row, while the AR2ns one Eq. (2) is in the bottom row. **(A)** original AR2s, **(B)** IAAFT surrogate and **(C)** PWIAAFT (ρ = 0.01) surrogate. **(D)** AR2ns Eq. (2) and one example of **(E)** IAAFT surrogate and **(F)** PWIAAFT (ρ = 0.01) surrogate. Small-size panels show 8 randomly selected segments of 50 data points obtained from the whole time series. The dashed lines represent the means and the dotted lines indicate one standard deviation. AR2s original, IAAFT and PWIAAFT surrogate series were identified as stationary. Whereas AR2ns original and PWIAAFT surrogate series were regarded as nonstationary, the corresponding IAAFT surrogate was identified as stationary. Time series in all panels are shown as arbitrary units.

**FIGURE 2:**
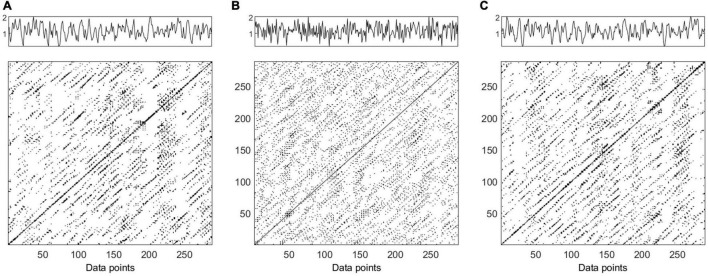
Time series and recurrence plots (*m* = 5, τ = 3) for synthetic data AR2s Eq. (1). Original time series **(panel A)**, one surrogate obtained by Iterative Amplitude Adjusted Fourier Transform (IAAFT) **(panel B)** and one surrogate obtained by Pinned Wavelet Iterative Amplitude Adjusted Fourier Transform (PWIAAFT) **(panel C)**. Time series in all panels are shown as arbitrary units.

**FIGURE 3:**
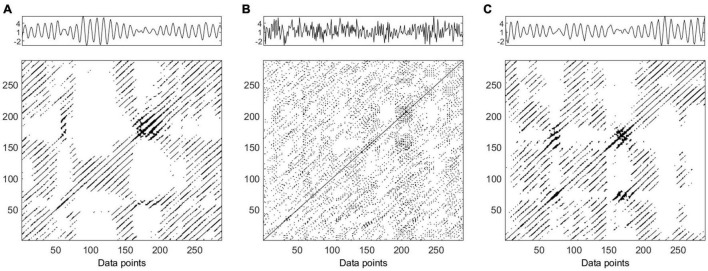
Time series and recurrence plot (*m* = 5, τ = 3) for synthetic data AR2ns Eq. (2). Original time series **(panel A)**, one surrogate obtained by Iterative Amplitude Adjusted Fourier Transform (IAAFT) **(panel B)** and one surrogate obtained by Pinned Wavelet Iterative Amplitude Adjusted Fourier Transform (PWIAAFT) **(panel C)**. Time series in all panels are shown as arbitrary units.

Figure 1 shows original data from the stationary second order autoregressive process (AR2s – panel A) and nonstationary second order autoregressive (AR2ns – panel D), which were appropriately identified as stationary and nonstationary by the restricted weak stationarity test, respectively (Section “Stationarity testing”). Illustrative examples of the stationarity testing as applied to IAAFT surrogates (middle column) and PWIAAFT (ρ = 0.01) surrogates (right column) are also shown in Figure 1. The IAAFT surrogates of both AR2s (panel B) and AR2ns (panel E) were regarded as stationary. In the PWIAAFT surrogate of AR2s (panel C), a restricted weak stationarity is detected, which was not identified in the PWIAAFT surrogate of AR2ns (panel F). These time series (original and surrogates) correspond to the time series shown in Figures 2, 3.

#### Nonlinearity Testing of AR2—Stationary

The AR2s original time series RP is shown in Figure 2 (panel A); the IAAFT algorithm applied to this series generated a noisy pattern in RP (panel B). The following RQA indices obtained from the IAAFT surrogates falsely rejected the null hypothesis: ADL, DET, ENT, LAM, LLDL, LLVL, RR, T2, and TT. On the other hand, the null hypothesis is accepted by considering RPDE, T1, CC, and TRANS. PWIAAFT surrogates with ρ = 0.01 (panel C) assessed with all RQA indices were found consistent with the null hypothesis. We also explored more PWIAAFT surrogates generated by ρ equal to 0, 0.03, and 0.1. In all cases, the same results were obtained.

#### Nonlinearity Testing of AR2 – Nonstationary

Figure 3 shows the RPs of the AR2ns original data (panel A), IAAFT surrogate (panel B), and PWIAAFT surrogate with ρ = 0.01 (panel C). The results of all RQA indices as applied to IAAFT surrogates were not in accordance with the null hypothesis. These results obtained from the PWIAAFT algorithm were consistent with the null hypothesis for all RQA indices. Other PWIAAFT surrogates generated with the parameter ρ of 0, 0.03 and 0.1 reflected the same findings.

**FIGURE 4:**
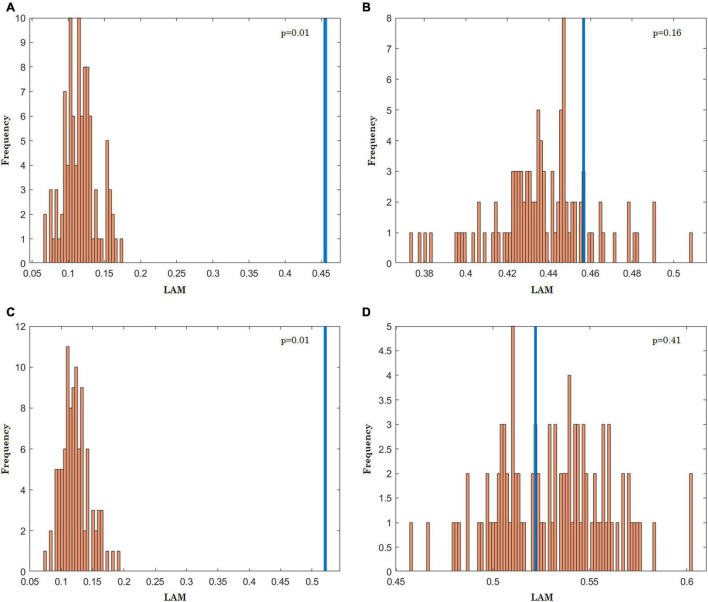
Histograms for the laminarity (LAM) values of recurrence plot from of 99 surrogates (orange) obtained with Iterative Amplitude Adjusted Fourier Transform (IAAFT) and Pinned Wavelet Iterative Amplitude Adjusted Fourier Transform (PWIAAFT) techniques. The LAM values measured from the original data are depicted in blue. AR2s, IAAFT **(A)**, PWIAAFT **(B)**; AR2ns, IAAFT **(C)**, PWIAAFT **(D)**.

An example of the distribution of a tested RQA statistic (LAM) for ARs and ARns is presented in Figure 4. Regarding IAAFT technique, *p* = 0.01 for both stationary and nonstationary linear processes, conversely, PWIAAFT surrogates accept the null hypothesis for both linear time series (*p* > 0.05).

### Heart Rate Variability Data

#### Time Domain and Spectral Heart Rate Variability Indices

The meanNN index was larger (lower heart rate) in supine position compared with active standing in the healthy group and ESRD patients after HD (Table 1). LF/HF was smaller in supine position compared with active standing in the healthy group. A larger meanNN value was observed in the healthy group compared to ESRD before HD and after HD in supine position. Also, meanNN was larger compared to ESRD after HD during active standing. SDNN was larger in healthy individuals compared to ESRD patients before and after HD in both positions. LF/HF ratio was larger in healthy individuals when compared to ESRD patients before HD, but this difference was not found when compared to ESRD patients after HD. During active standing, LF/HF was different between ESRD patients before and after HD.

**TABLE 1:** Time domain and spectral heart rate variability (HRV) indices shown as median values (95% confidence interval of the median).

			ESRD group			
	Healthy group (*N* = 40)		Before HD (*N* = 29)		After HD (*N* = 29)	
	Supine	Standing	Supine	Standing	Supine	Standing
meanNN (s)	0.897* (0.845–0.927)	0.719 (0.678–0.752)	0.729^§^ (0.670–0.824)	0.686 (0.644–0.750)	0.674*^¶^ (0.643–0.837)	0.569^¶^ (0.540–0.669)
SDNN (s)	0.050 (0.046–0.063)	0.040 (0.036–0.046)	0.024^§^ (0.015–0.025)	0.027^§^ (0.020–0.033)	0.025^¶^ (0.016–0.032)	0.019^¶^ (0.015–0.027)
LF/HF	1.338* (1.033–1.685)	4.417 (3.112–6.050)	2.942^§^ (1.837–4.046)	4.741° (2.450–7.308)	2.240 (1.506–3.996)	4.390 (2.850–7.525)

*p < 0.005 supine vs. standing.

*^§^p < 0.005 before HD vs. healthy (same position).*

*^¶^p < 0.005 after HD vs. healthy (same position).*

*°p < 0.005 before HD vs. after HD (same position).*

#### Stationarity Testing

**FIGURE 5:**
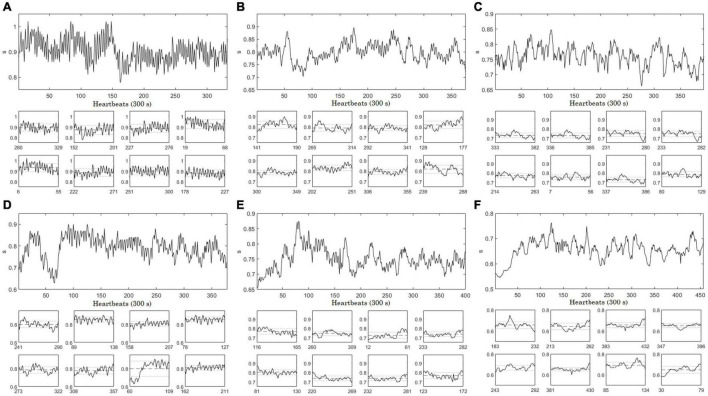
Examples of nonstationary heart rate variability (HRV) time series. Full-size panels show the whole HRV time series in supine position (top row) of **(A)** healthy subject, **(B)** end stage renal disease (ESRD) patient before hemodialysis (HD), and **(C)** ESRD patient after HD (same individual). HRV time series collected at active standing (bottom row) from **(D)** healthy subject, **(E)** ESRD patient before HD, and **(F)** ESRD patient after HD. HRV time series units in all panels are shown as seconds (s).

Figure 5 shows examples of stationary testing applied to HRV data in supine position (top row) and active standing (bottom row) from a healthy subject (left column), an ESRD patient before HD (middle column) and an ESRD patient after HD (right column). All the examples shown in Figure 5 were classified as nonstationary.

The original HRV time series were mostly classified as nonstationary; only 3 of the 196 analyzed time series were identified as stationary (about 1.5%). The 3 stationary HRV time series were obtained from a healthy subject (supine position), an ESRD patient after HD (supine position), and an ESRD patient after HD (active standing).

#### Surrogate Data Testing

**FIGURE 6:**
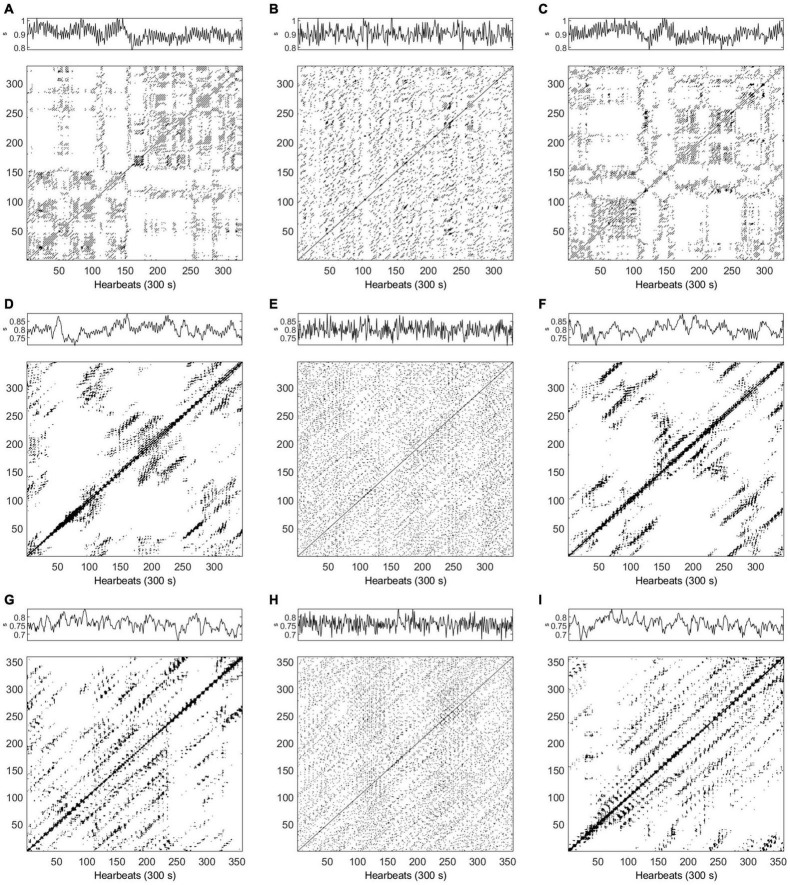
**(A–I)** Examples of time series and recurrence plots for heart rate variability (HRV) data in supine position. Top row corresponds to a healthy subject (*m* = 4, τ = 1), **(A)** original data, **(B)** Iterative Amplitude Adjusted Fourier Transform (IAAFT) surrogate, and **(C)** Pinned Wavelet Iterative Amplitude Adjusted Fourier Transform (PWIAAFT) surrogate. Middle row, end stage renal disease (ESRD) patient before hemodialysis (HD) (*m* = 6, τ = 6), **(D)** original data, **(E)** IAAFT surrogate, and **(F)** PWIAAFT surrogate. Bottom row, ESRD patient after HD (*m* = 6, τ = 7), **(G)** original data, **(H)** IAAFT surrogate, and **(I)** PWIAAFT surrogate. HRV time series units in all panels are shown as seconds (s).

**FIGURE 7:**
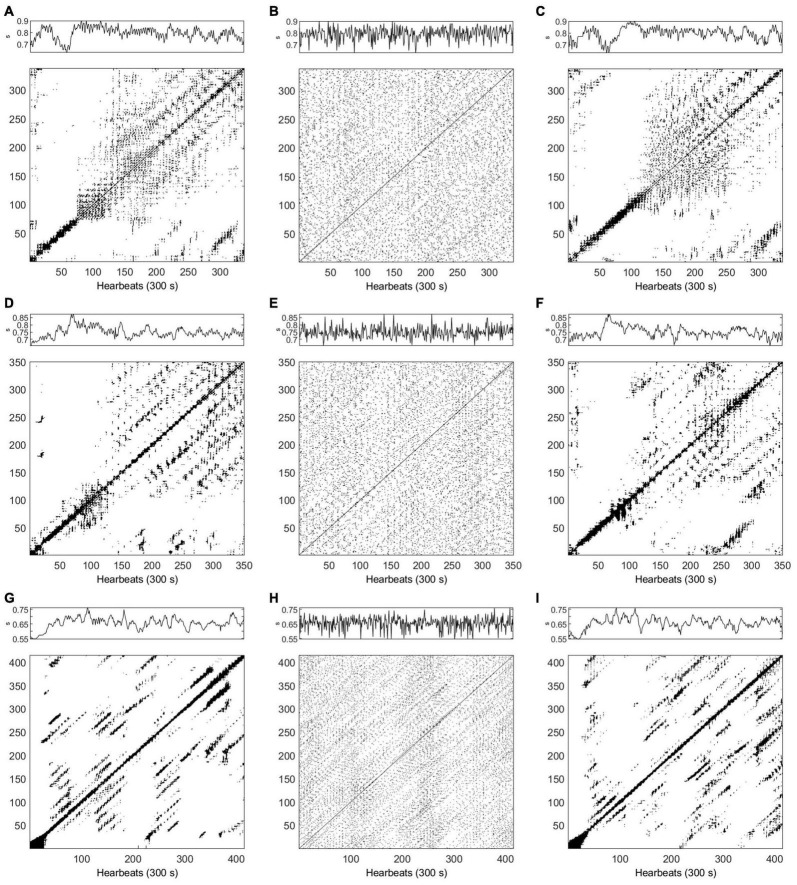
**(A–I)** Examples of time series and recurrence plots for heart rate variability (HRV) data in active standing. Top row corresponds to a healthy subject (*m* = 5, τ = 10), **(A)** original data, **(B)** Iterative Amplitude Adjusted Fourier Transform (IAAFT) surrogate, and **(C)** Pinned Wavelet Iterative Amplitude Adjusted Fourier Transform (PWIAAFT) surrogate. Middle row, end stage renal disease (ESRD) patient before hemodialysis (HD) (*m* = 6, τ = 10), **(D)** original data, **(E)** IAAFT surrogate, and **(F)** PWIAAFT surrogate. Bottom row, ESRD patient after HD (*m* = 8, τ = 6), **(G)** original data, **(H)** IAAFT surrogate, and **(I)** PWIAAFT surrogate. HRV time series units in all panels are shown as seconds (s).

Examples of HRV time series of healthy and ESRD subjects (before and after HD), RP and corresponding surrogates in supine position and active standing are displayed in Figures 6, 7, respectively (the same examples shown in Figure 5). While the recurrence points are dispersed over all the RP in the IAAFT surrogates (middle column), PWIAAFT surrogates (right column) provide a similar distribution of recurrence points compared to the original time series (left column). This is observed for healthy subjects and ESRD patients before and after HD in both supine position (Figure 6) and active standing (Figure 7). Recurrence quantitative analysis indices in almost all IAAFT surrogates lead to reject the null hypothesis (Table 2). However, in comparison the number of cases with null hypothesis rejections (the percentage of time series in which the surrogate data testing null hypothesis was rejected) was significantly lower using the PWIAAFT surrogates, with exception of CC and TRANS. Although the results of PWIAAFT surrogates shown in Figures 1–7 and Table 2 were generated with ρ = 0.01, we also explored the following values: 0.00, 0.03, and 0.10. We did not find statistically different proportions of rejection rates using these values (Supplementary Table 1).

**TABLE 2:** Percentage (95% confidence Interval) of heart rate variability (HRV) time series in every group that reject the null hypothesis according to the results of different recurrence quantitative analysis (RQA) indices.

			ESRD group			
IAAFT	Healthy group (*N* = 40)		Before HD (*N* = 29)		After HD (*N* = 29)	
	Supine	Standing	Supine	Standing	Supine	Standing
RR	65 (49.6–78.3)	97.5 (88.9–99.7)	89.7 (74.9–97)	93.1 (79.7–98.5)	93.1 (79.7–98.5)	100
DET	100	100	100	100	100	100
ADL	100	100	100	100	100	100
LLDL	82.5 (68.7–91.8)	97.5 (88.9–99.7)	100	96.6 (85–99.6)	93.1 (79.7–98.5)	96.6 (85–99.6)
ENT	100	100	100	100	100	100
TT	80 (65.8–90.1)	97.5 (88.9–99.7)	93.1 (79.7–98.5)	93.1 (79.7–98.5)	89.7 (74.9–97)	100
LLVL	70 (54.8–82.4)	97.5 (88.9–99.7)	93.1 (79.7–98.5)	93.1 (79.7–98.5)	89.7 (74.9–97)	100
T1	75 (60.2–86.4)	80 (65.8–90.1)	93.1 (79.7–98.5)	82.8 (66.3–93.1)	82.8 (66.3–93.1)	93.1 (79.7–98.5)
T2	85 (71.7–93.5)	100	96.6 (85–99.6)	100	0.966 (85–99.6)	100
RPDE	80 (65.8–90.1)	55 (39.7–69.6)	75.9 (58.4–88.5)	58.6 (40.6–75)	72.4 (54.6–86)	62.1 (44–77.9)
CC	65 (49.6–78.3)	77.5 (62.9–88.2)	89.7 (74.9–97)	86.2 (70.5–95.2)	86.2 (70.5–95.2)	75.9 (58.4–88.5)
TRANS	65 (49.6–78.3)	75 (60.2–86.4)	89.7 (74.9–97)	89.7 (74.9–97)	75.9 (58.4–88.5)	72.4 (54.6–86)

			**ESRD group**			
			
**PWIAAFT**	**Healthy group (*N* = 40)**		**Before HD (*N* = 29)**		**After HD (*N* = 29)**	
			
	**Supine**	**Standing**	**Supine**	**Standing**	**Supine**	**Standing**

RR	22.5 (11.8–37.1) *	25 (13.6–39.8) *	24.1 (11.5–41.6) *	20.7 (9.1–37.8) *	17.2 (6.9–33.7) *	20.7 (9.1–37.8) *
DET	60 (44.6–74.1) *	27.5 (15.6–42.5) *	31 (16.6–49) *	34.5 (19.3–52.6) *	37.9 (22.1–56) *	34.5 (19.3–52.6) *
ADL	55 (39.7–69.6) *	32.5 (19.6–47.8) *	41.4 (25–59.4) *	31 (16.6–49) *	31 (16.6–49) *	24.1 (11.5–41.6) *
LLDL	15 (6.5–28.3) *	10 (3.5–22) *	20.7 (9.1–37.8) *	17.2 (6.9–33.7) *	13.8 (4.8–29.5) *	10.3 (3–25.1) *
ENT	50 (35–65) *	37.5 (23.8–52.9) *	41.4 (25–59.4) *	31 (16.6–49) *	24.1 (11.5–41.6) *	24.1 (11.5–41.6) *
TT	47.5 (32.7–62.7) *	25 (13.6–39.8) *	41.4 (25–59.4) *	31 (16.6–49) *	34.5 (19.3–52.6) *	20.7 (9.1–37.8) *
LLVL	20 (9.9–34.2) *	0.0*	10.3 (3–25.1) *	0.0*	6.9 (1.5–20.3) *	3.4 (0.4–15) *
T1	32.5 (19.6–47.8) *	22.5 (11.8–37.1) *	24.1 (11.5–41.6) *	17.2 (6.9–33.7) *	13.8 (4.8–29.5) *	20.7 (9.1–37.8) *
T2	45 (30.4–60.3) *	15 (6.5–28.3) *	27.6 (14–45.4) *	24.1 (11.5–41.6) *	13.8 (4.8–29.5) *	34.5 (19.3–52.6) *
RPDE	35 (21.7–50.4) *	10 (3.5–22) *	31 (16.6–49) *	10.3 (3–25.1) *	13.8 (4.8–29.5) *	17.2 (6.9–33.7) *
CC	42.5 (28.1–57.9)	20 (9.9–34.2)	27.6 (14–45.4)	6.9 (1.5–20.3)	34.5 (19.3–52.6)	34.5 (19.3–52.6)
TRANS	42.5 (28.1–57.9)	20 (9.9–34.2)	34.5 (19.3–52.6)	10.3 (3–25.1)	31 (16.6–49)	34.5 (19.3–52.6)

*p < 0.001 PWIAAFT vs. IAAFT (same group and position).

**FIGURE 8:**
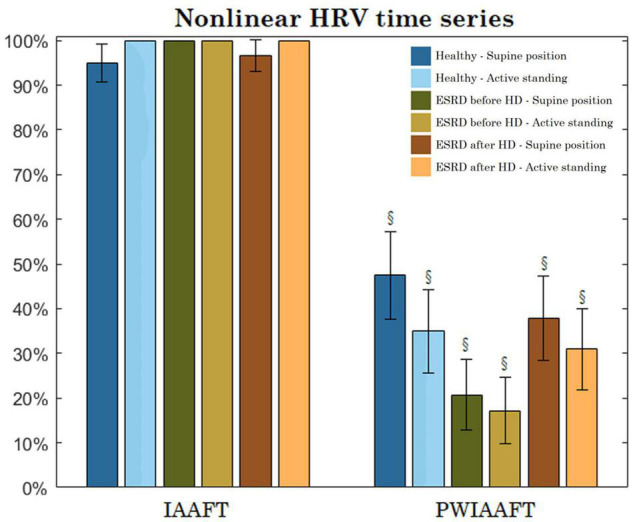
Percentage of heart rate variability (HRV) time series of every group that leads to reject the null hypothesis (nonlinearity demonstrated) using Iterative Amplitude Adjusted Fourier Transform (IAAFT) and Pinned Wavelet Iterative Amplitude Adjusted Fourier Transform (PWIAAFT) techniques (bars display the 95% confidence interval). § PWIAAFT vs IAAFT in the same group *p* < 0.001. There were no significant differences between groups (same position) nor within groups (supine vs active standing, same group).

Figure 8 shows the percentage of nonlinear time series using LAM statistic. A trend toward lower rejection rates was found in ESRD patients before hemodialysis compared with healthy subjects; this trend can be observed in active standing compared to supine position. However, no statistically significant differences were found. The following rejection rates of LAM correspond to IAAFT surrogates: healthy group supine position 95% (90.72%–99.27%, CI 95%), active standing 100%. End stage renal disease group before HD at supine position and active standing 100% rejections; ESRD group after HD at supine position 96.6% (93.04%–99.9%, CI 95%) and active standing 100%. Rejections rates of LAM with PWIAAFT surrogates were: healthy group at supine position 47.5% (37.71%–57.28%, CI 95%) and active standing 35% (25.65%–44.34%, CI 95%). End stage renal disease group before HD at supine position 20.7% (12.75%–28.64%, CI 95%) and active standing 17.2% (9.8%–24.59%, CI 95%) rejections; ESRD group after HD at supine 37.9% (28.39%–47.4%, CI 95%) and standing 31% (21.93%–40.06%, CI 95%) rejections. The trend toward lower rejection rates in ESRD was also observed in other RQA indices (i.e., DET, ENT, LLVL, TT) (Table 2).

#### Correlations With meanNN

The meanNN index is linearly correlated with embedding parameters in healthy subjects, as it is shown in Table 3. This correlation with *m* is lost in ESRD patients before hemodialysis; however, it is regained after hemodialysis treatment. The meanNN index is also correlated with many of RQA indices in original data (Table 4). These correlations are lost in most parameters (except for LAM, TT, and LLVL) in ESRD patients before hemodialysis. After treatment, meanNN is significantly correlated with all RQA indices.

**TABLE 3:** Spearman correlation coefficient between meanNN and the embedding parameters, tau (τ) and dimension (*m*).

			ESRD group			
	Healthy group (*N* = 40)	p	Before HD (*N* = 29)	p	After HD (*N* = 29)	p
τ	−0.478	< 0.001	–0.323	0.013	–0.522	< 0.001
*m*	–0.237	0.034	–0.222	0.094	–0.402	0.002

**TABLE 4:** Spearman correlation coefficients between meanNN and recurrence quantitative analysis (RQA) indices for the original heart rate variability (HRV) time series.

			ESRD group			
RQA	Healthy group	p	Before HD	p	After HD	p
index	(*N* = 40)		(*N* = 29)		(*N* = 29)	
RR	0.396	<0.001	0.141	0.292	0.451	<0.001
DET	–0.012	0.913	–0.254	0.054	–0.268	0.042
ADL	0.133	0.24	–0.045	0.736	–0.39	0.003
LLDL	–0.276	0.013	–0.027	0.839	–0.418	0.001
ENT	0.165	0.144	–0.069	0.606	–0.388	0.003
LAM	–0.523	<0.001	–0.284	0.031	–0.422	0.001
TT	–0.593	<0.001	–0.38	0.003	–0.643	<0.001
LLVL	–0.643	<0.001	–0.546	<0.001	–0.68	<0.001
T1	–0.075	0.51	–0.001	0.992	0.335	0.01
T2	–0.536	<0.001	–0.239	0.071	–0.308	0.019
RPDE	–0.244	0.029	0.108	0.421	0.499	<0.001
CC	0.389	<0.001	0.199	0.135	0.508	<0.001
TRANS	0.375	0.001	0.202	0.128	0.523	<0.001

In the same manner as above, we assessed the correlation with meanNN and mean values of RQA in both IAAFT (Table 5) and PWIAAFT (ρ = 0.01) surrogates (Table 6). Regarding IAAFT surrogates, the meanNN is correlated only with RR, T1, and RPDE in the healthy group. In ESRD patients before hemodialysis, meanNN was correlated only with LLDL, LLVL, T2, and RPDE. But after treatment, meanNN was correlated with almost all RQA indices, with the exception of ADL, TT, T1 and T2. Using the PWIAAFT surrogates, we found better preservation in comparison with IAAFT of the correlation between meanNN and RQA indices, as observed in Table 5.

**TABLE 5:** Spearman correlation coefficients between meanNN and recurrence quantitative analysis (RQA) indices for Iterative Amplitude Adjusted Fourier Transform (IAAFT) surrogates of heart rate variability (HRV) time series.

			ESRD group			
RQA	Healthy group	p	Before HD	p	After HD	p
index	(*N* = 40)		(*N* = 29)		(*N* = 29)	
RR	–0.345	0.002	–0.115	0.389	–0.45	<0.001
DET	–0.052	0.649	–0.242	0.068	–0.466	<0.001
ADL	0.133	0.239	–0.039	0.771	–0.243	0.066
LLDL	0.204	0.07	–0.353	0.007	–0.619	<0.001
ENT	0.102	0.367	–0.081	0.545	–0.385	0.003
LAM	–0.054	0.635	–0.201	0.131	–0.414	0.001
TT	0.133	0.241	0.036	0.791	–0.096	0.474
LLVL	–0.025	0.825	–0.427	0.001	–0.615	<0.001
T1	–0.268	0.016	–0.233	0.079	–0.207	0.118
T2	–0.131	0.247	–0.295	0.025	–0.208	0.118
RPDE	0.615	<0.001	0.349	0.007	0.728	<0.001
CC	0.196	0.081	0.174	0.191	0.28	0.031
TRANS	0.17	0.131	0.159	0.232	0.26	0.049

**TABLE 6:** Spearman correlation coefficients between meanNN and recurrence quantitative analysis (RQA) indices for Pinned Wavelet Iterative Amplitude Adjusted Fourier Transform (PWIAAFT) (ρ = 0.01) surrogate data of heart rate variability (HRV) time series.

			ESRD group			
RQA	Healthy group	p	Before HD	p	After HD	p
index	(*N* = 40)		(*N* = 29)		(*N* = 29)	
RR	0.394	<0.001	0.148	0.267	0.478	<0.001
DET	–0.05	0.658	–0.257	0.052	–0.311	0.017
ADL	0.091	0.422	–0.108	0.42	–0.42	0.001
LLDL	–0.349	0.001	–0.148	0.269	–0.459	<0.001
ENT	0.122	0.28	–0.114	0.396	–0.429	0.001
LAM	–0.583	<0.001	–0.273	0.038	–0.442	0.001
TT	–0.656	<0.001	–0.407	0.002	–0.665	<0.001
LLVL	–0.811	<0.001	–0.607	<0.001	–0.79	<0.001
T1	–0.078	0.49	0.021	0.877	0.138	0.303
T2	–0.588	<0.001	–0.271	0.04	–0.437	0.001
RPDE	–0.047	0.679	0.176	0.187	0.539	<0.001
CC	0.391	<0.001	0.214	0.127	0.589	<0.001
TRANS	0.391	<0.001	0.187	0.161	0.498	<0.001

## Discussion

### Contribution

We show the application of RQA indices as discriminative nonlinear statics in surrogate data testing and proved the presence of nonlinear structures in short-term HRV time series of healthy subjects and ESRD patients during an active standing test. Other contribution of this work is the implementation of PWIAAFT surrogates for the analysis of HRV data. This method facilitates nonlinear testing as the *a priori* demonstration of stationarity is not strictly needed. This condition is rarely identified in HRV data (Niccolai et al., 1995; Braun et al., 1998; Porta et al., 2004; Gao et al., 2013), particularly if these data are obtained from healthy subjects studied during daily or ambulatory conditions. Our findings show that even in controlled scenarios, most of healthy subjects and ESRD patients exhibit nonstationary behavior.

Recurrence quantitative analysis (RQA) has been widely used for assessing HRV data, its advantages for the analysis of short, noisy and nonstationary time series becomes a convenient feature for the study of cardiovascular physiology (Marwan et al., 2002). However, nonlinearity by itself, to our best knowledge has not been tested by means of RQA in short-term HRV recordings. Surrogate data testing is a well-known procedure to prove nonlinearity by contradiction. However, the presence of a nonstationary behavior may become a limitation to obtain either reliable HRV indices, such as those provided by the frequency domain analysis (Li et al., 2019), or even appropriate surrogates.

### Synthetic Data

In this work we applied the IAAFT technique to linear synthetic stationary and real nonstationary data. It has been suggested that IAAFT surrogates lead to falsely accept the null hypothesis due to their small deviations of the applied statistic measure and rigid preservation of the linear properties in time series (Lancaster et al., 2018). However, some recurrence indices applied here lead to falsely reject the null hypothesis in stationary linear synthetic data. This finding may indicate that RQA is particularly sensible to the randomization of the data and rupture of their structure. In nonstationary synthetic time-series, all statistic measures falsely rejected the null hypothesis, even those that adequately lead to accept the linear hypothesis of stationary data. It is known that “stationarization” (the introduction of stationarity in the timeseries) is a property of surrogates obtained by the IAAFT technique and this may be a reason for higher false rejections (Borgnat and Flandrin, 2009; Lancaster et al., 2018). It is important to emphasize that this technique can lead to null hypothesis rejections because the original time series are either nonlinear or by contrast nonstationary. This phenomenon was previously observed in HRV time series using other discriminative statistics (Faes et al., 2009), finding that the actual rate of rejections decreases once that the technique for surrogate data generation considers nonstationarity. PWIAAFT takes into consideration this characteristic and preserves accurately the original linear structure of the data, as it is shown in this work, for both stationary and non-stationary data. This technique allowed the acceptance of the null hypothesis with all the RQA indices as applied to linear synthetic data.

### Heart Rate Variability Data

Traditional HRV indices (Table 1) show the increased sympathetic predominance associated to active standing and ESRD (Gonzalez et al., 2013; Gonzalez-Gomez et al., 2018; Calderon-Juarez et al., 2020). Regarding nonlinear testing of HRV, in a previous study using data generated through Fourier transform-based surrogates (i.e., IAAFT) (Porta et al., 2007), a very low proportion of short-term HRV time series from healthy subjects was found to be nonlinear. But these series are not intuitively expected to be linear due to the nonlinear mechanisms modulating heart rate that are generally considered to be involved. It is possible therefore that such series in that study were too noisy or too short to clearly exhibit nonlinear dynamics. In addition, the activation of the sympathetic branch of the autonomic nervous system decreases the proportion of nonlinear time series, this has been corroborated by pharmacological stimulation and the gradual head-up tilt test (Porta et al., 2007). It has also been suggested that cardiorespiratory coupling confers nonlinear behavior to HRV, because the controlled respiration at a slow rate introduce nonlinear dynamics to HRV (Porta et al., 2000).

In this work, when the IAAFT surrogates were obtained from HRV data, a high rate of null hypothesis was confirmed in relation to RQA indices. Nonetheless, the results for synthetic data demonstrate that these findings can be misleading. Furthermore, only approximately 1.5% of all the HRV time series analyzed in this work were regarded as stationary. As explained above, this is a potential source leading to false nonlinearity detections. It is remarkable that PWIAAFT surrogates show an important decrease in the rate of rejection, similarly to the results shown by time-varying autoregressive surrogate series (Faes et al., 2009), which also involve nonstationary behavior. Added to the well-known PWIAAFT conservation of nonstationarity (Keylock, 2007, 2019; Keylock et al., 2011, 2015) and the ubiquitous presence of nonstationarity in the analyzed HRV time series, the dramatic drop of nonlinearity detection shown by PWIAAFT in comparison to IAAFT is thus likely related to the elimination of the instability of mean and variance in the IAAFT surrogates.

Depending on the RQA index, the percentage of short-term HRV recordings that are found to contain nonlinear properties can be as high as 60% in healthy subjects when DET is used as the statistic measure. For the ESRD patients, the rejection rate decreases to 31% before HD treatment and 34.5% after HD. Furthermore, this rejection rate tends to even lower values in active standing compared with supine position for healthy and ESRD patients, but there were not statistically significant differences regarding this position. These findings suggest that RQA is a suitable tool to detect nonlinearity in short-term series, even when these series manifest nonstationarity. Other pathophysiological conditions, such as acute myocardial infarction have been addressed (Faes et al., 2019) with the surrogate data approach. Patients with this condition tend to show lower proportions of nonlinear HRV times series, which is similar to ESRD patients studied in this work. All these findings suggest that some pathologies suppress nonlinearity from HRV dynamics.

It was proposed by a previous work (Calderon-Juarez et al., 2020) that the meanNN parameter as obtained from HRV data is linearly correlated with some RQA indices in healthy subjects. Notwithstanding that the underlying physiological mechanism of these correlations is not clearly known, an intricate multilayer of physiological interactions could be involved (Kooman et al., 2018). As previously identified (Calderon-Juarez et al., 2020), these correlations are known to be lost in ESRD patients and partially retrieved after hemodialysis. The correlations between meanNN and RQA indices are no longer present in IAAFT surrogates probably owing to the poor conservation of the original time series structure. Yet most of these correlations are preserved with the PWIAAFT surrogates, suggesting that these correlations are partially given by linear statistical and spectral parameters. Some authors have proposed to normalize HRV linear indices by dividing them with the mean heart rate to correct, by this approach, the influence of heart rate on HRV (Hayano et al., 1991). Monfredi et al. (2014) have also shown a robust correlation of mean heart rate and standard deviation of NN intervals; however, they claim that such normalization is insufficient to adequately correct the nonlinear influence of heart rate on HRV (Monfredi et al., 2014). Our work shows that the surrogates HRV time series, in which any nonlinearity structure is destroyed, such correlation of the mean heart rate with RQA is preserved. Notwithstanding that other factors such as age and sex also modify HRV, the meanNN is a determinant characteristic of these time series because it explains a significant dispersion of the RQA indices, thus these indices could also be subjected to normalization by the meanNN.

### Limitations and Perspectives

The study of several types of nonlinear behaviors and other types of nonstationarities is beyond the scope of this work. Further research may be conducted to identify which RQA indices are suitable for testing different nonlinear structures. As proposed by Borgnat and Flandrin (2009), nonstationarity can be in fact tested by the generation of stationary surrogate data, which may be considered for future studies of HRV data. Longer HRV time series, which contain enough information to address slower fluctuations and therefore pose different physiological mechanisms of regulation (Lerma et al., 2017), were not explored in this work and these series should be assessed in future projects as well. We collected a small number of ESRD and active standing recordings, thus any potential lower rate of null hypothesis rejections for these data was not possible to be addressed. The respiratory cycle is another physiological factor that influences the HRV time series, its effect remains to be assessed with the combination of techniques presented here. Future studies are required to assess the nonlinear behavior with other HRV indices that are assumed to reflect nonlinearity and to compare them with the present findings.

## Conclusion

Recurrence quantitative analysis (RQA) is a suitable framework for the analysis of short, noisy, nonstationary time series and here we also endorse that it is sensitive to capture nonlinear features despite the drawbacks in physiological data analysis that can be introduced by ubiquitous conditions such as the nonstationary behavior. We found that an important proportion of HRV time series from healthy subjects and ESRD patients do contain nonlinear information and hence may be studied from a nonlinear scope point of view to achieve a broader understanding of cardiovascular physiology.

## Data Availability Statement

The raw data supporting the conclusions of this article will be made available upon request to the corresponding author, provided pertinent legal requirements are met.

## Ethics Statement

The studies involving human participants were reviewed and approved by Research and Ethics Committee of the Instituto Nacional de Cardiología Ignacio Chávez (protocol number 21-1236). The patients/participants provided their written informed consent to participate in this study.

## Author Contributions

MC-J, GG, JE, and CL: conceptualization, methodology, and writing—original draft preparation. MC-J and CL: software. GG, HP-G, and CL: resources. MC-J, GG, EQ, and CL: data gathering. MC-J, GG, JE, HP-G, and CL: writing—review and editing. GG: funding acquisition. All authors have read and agreed to the published version of the manuscript.

## Conflict of Interest

The authors declare that the research was conducted in the absence of any commercial or financial relationships that could be construed as a potential conflict of interest.

## Publisher’s Note

All claims expressed in this article are solely those of the authors and do not necessarily represent those of their affiliated organizations, or those of the publisher, the editors and the reviewers. Any product that may be evaluated in this article, or claim that may be made by its manufacturer, is not guaranteed or endorsed by the publisher.

## Appendix A

Recurrence quantitative analysis indices computed by Cross Recurrence Plot Toolbox for MATLAB.

Recurrence rate (RR) (Marwan et al., 2007)

$$R\,R=\frac{1}{N^{2}}\,\sum_{i,j=1}^{N}R_{i,j}$$

Determinism (DET) (Marwan et al., 2007)

$$D\,E\,T=\frac{\sum_{l=l_{m\,i\,n}}^{N}l\,P^{\in }\,\left(l\right)}{\sum_{i,j}^{N}R_{i,j}}$$

(where *P*^∈^ (*l*) = {*l*_*i*_; *i* = 1…*N*_*i*_} is the frequency distribution of the lengths *l* of diagonal structures and *N*_*l*_ is the absolute number of diagonal lines).

Averaged diagonal length (ADL) (Marwan et al., 2007)

$$A\,D\,L=\frac{\sum_{l_{i=1}}^{l_{N}}l_{i}}{N}$$

Length of longest diagonal line (LLDL) (Marwan et al., 2007)

$$LLDL=max\,\left(\left\{l_{i};i=1\ldots N_{i}\right\}\right)$$

Entropy of diagonal line, Shannon’s entropy (ENT) (Marwan et al., 2007)

$$E\,N\,T=-\sum_{l=l_{m\,i\,n}}^{N}p\,\left(l\right)\,l\,n\,p\,\left(l\right)$$

Laminarity (LAM) (Marwan et al., 2002)

$$L\,A\,M=\frac{\sum_{v=v_{m\,i\,n}}^{N}v\,P^{\varepsilon }\,\left(v\right)}{\sum_{v=1}^{N}v\,P^{\varepsilon }\,\left(v\right)}$$

(where *P*^ε^(*v*) = {*v*_*i*_; *i* = 1…*N*_*v*_} denotes the frequency distribution of the *l* lengths of vertical structures).

Trapping time (TT) (Marwan et al., 2002)

$$T\,T=\frac{\sum_{v=v_{m\,i\,n}}^{N}v\,P^{\varepsilon }\,\left(v\right)}{\sum_{v=v_{m\,i\,n}}^{N}P^{\varepsilon }\,\left(v\right)}$$

Length of longest vertical line (LLVL) (Marwan et al., 2002)

$$LLVL=max\left(\left\{v_{i};i=1\ldots N_{i}\right\}\right)$$

Recurrence time of the 1*^st^* type (T1) (Gao and Cai, 2000)

$$T_{j}^{1}=\left|\left\{i,j:{\vec{x}}_{i},{\vec{x}}_{j}\in R_{i}\right\}\right|$$

Recurrence time of the 2*^nd^* type (T2) (Gao and Cai, 2000)

$$T_{j}^{2}=\left|\left\{i,j:{\vec{x}}_{i},{\vec{x}}_{j}\in R_{i};{\vec{x}}_{j-1}\notin R_{i}\right\}\right|$$

(where *R*_*i*_ are the recurrence points which belong to the state ${\vec{x}}_{i}$).

Recurrence period density entropy (RPDE) (Little et al., 2007)

$$H_{n\,o\,r\,m}=\frac{-\sum_{i=1}^{T_{m\,a\,x}}P\,\left(i\right)\,l\,n\,P\,\left(i\right)}{l\,n\,T_{m\,a\,x}}$$

(where *P*(*i*) is the recurrence period density, *T*_*max*_ is the maximum recurrence time found in the embedded state space).

Clustering coefficient (CC) (Marwan et al., 2009)

$$C\,C=\sum_{i=1}^{N}\frac{\sum_{i,j,k=1}^{N}R_{i,j}\,R_{j,k}\,R_{k,i}}{R\,R_{i}}$$

Transitivity (TT) (Donner et al., 2010)

$$T\,R\,A\,N\,S=\sum_{i=1}^{N}\frac{\sum_{i,j,k=1}^{N}R_{i,j}\,R_{j,k}\,R_{k,i}}{\sum_{i,j,k=1}^{N}R_{i,j}\,R_{k,i}}$$
