# Supplementary material for: Recurrence Quantitative Analysis of Wavelet-Based Surrogate Data for Nonlinearity Testing in Heart Rate Variability
Source: Front Physiol. 2022 Feb 9;13:807250. doi: 10.3389/fphys.2022.807250 (PMC8864246; doi:10.3389/fphys.2022.807250)
Supplement: Supplementary file 1 [file Table_1.DOCX]

Supplementary Material

# Supplementary Table

Table S1. Percentage of null hypothesis rejections of HRV time series using surrogates from the Pinned Wavelet Iterative Amplitude Adjusted Fourier Transform (PWIAAFT) technique with different ρ values. HRV time series included data collected at supine and active standing from healthy subjects and End-Stage Renal Disease patients before hemodialysis (HD) and after hemodialysis. Parameter ρ is shown.

|  |  | Percentage of null hypothesis rejections | | | | | |
| --- | --- | --- | --- | --- | --- | --- | --- |
|  |  |  |  | ESRD group | | | |
|  |  | Healthy group (N=40) | | Before HD (N=29) | | After HD (N=29) | |
| Recurrence Quantitative Analysis index | Technique (ρ) | Supine | Standing | Supine | Standing | Supine | Standing |
| Recurrence rate (RR) |  |  |  |  |  |  |  |
|  | PWIAAFT (0) | 20% | 28% | 24% | 21% | 21% | 24% |
|  | PWIAAFT (0.03) | 23% | 23% | 28% | 14% | 17% | 24% |
|  | PWIAAFT (0.1) | 20% | 13% | 21% | 10% | 7% | 24% |
| Determinism (DET) |  |  |  |  |  |  |  |
|  | PWIAAFT (0) | 58% | 33% | 34% | 31% | 38% | 31% |
|  | PWIAAFT (0.03) | 53% | 28% | 31% | 34% | 38% | 34% |
|  | PWIAAFT (0.1) | 50% | 28% | 31% | 45% | 34% | 24% |
| Averaged diagonal length (ADL) |  |  |  |  |  |  |  |
|  | PWIAAFT (0) | 55% | 30% | 41% | 34% | 28% | 21% |
|  | PWIAAFT (0.03) | 58% | 35% | 41% | 34% | 31% | 21% |
|  | PWIAAFT (0.1) | 50% | 35% | 41% | 34% | 28% | 24% |
| Length of longest diagonal line (LLDL) |  |  |  |  |  |  |  |
|  | PWIAAFT (0) | 8% | 8% | 21% | 14% | 10% | 7% |
|  | PWIAAFT (0.03) | 18% | 8% | 24% | 14% | 21% | 3% |
|  | PWIAAFT (0.1) | 10% | 8% | 21% | 21% | 10% | 10% |
| Entropy of diagonal length (ENT) |  |  |  |  |  |  |  |
|  | PWIAAFT (0) | 50% | 28% | 41% | 31% | 17% | 21% |
|  | PWIAAFT (0.03) | 45% | 30% | 38% | 31% | 24% | 21% |
|  | PWIAAFT (0.1) | 48% | 30% | 34% | 31% | 21% | 24% |
| Laminarity (LAM) |  |  |  |  |  |  |  |
|  | PWIAAFT (0) | 48% | 35% | 21% | 17% | 38% | 31% |
|  | PWIAAFT (0.03) | 55% | 25% | 21% | 21% | 38% | 34% |
|  | PWIAAFT (0.1) | 45% | 33% | 24% | 24% | 41% | 28% |
| Trapping time (TT) |  |  |  |  |  |  |  |
|  | PWIAAFT (0) | 48% | 23% | 38% | 28% | 31% | 17% |
|  | PWIAAFT (0.03) | 40% | 23% | 41% | 24% | 31% | 17% |
|  | PWIAAFT (0.1) | 43% | 23% | 38% | 31% | 24% | 21% |
| Length of longest vertical line (LLVL) |  |  |  |  |  |  |  |
|  | PWIAAFT (0) | 5% | 3% | 3% | 0% | 3% | 3% |
|  | PWIAAFT (0.03) | 5% | 5% | 14% | 3% | 7% | 3% |
|  | PWIAAFT (0.1) | 10% | 3% | 3% | 3% | 3% | 3% |
| Recurrence time of 1st type (T1) |  |  |  |  |  |  |  |
|  | PWIAAFT (0) | 23% | 20% | 21% | 21% | 10% | 17% |
|  | PWIAAFT (0.03) | 25% | 13% | 24% | 17% | 7% | 17% |
|  | PWIAAFT (0.1) | 23% | 15% | 14% | 21% | 10% | 14% |
| Recurrence time of 2nd type (T2) |  |  |  |  |  |  |  |
|  | PWIAAFT (0) | 40% | 23% | 31% | 28% | 24% | 41% |
|  | PWIAAFT (0.03) | 40% | 18% | 24% | 24% | 14% | 31% |
|  | PWIAAFT (0.1) | 35% | 18% | 17% | 24% | 17% | 28% |
| Recurrence period density entropy (RPDE) |  |  |  |  |  |  |  |
|  | PWIAAFT (0) | 38% | 10% | 28% | 14% | 14% | 17% |
|  | PWIAAFT (0.03) | 33% | 15% | 28% | 10% | 14% | 14% |
|  | PWIAAFT (0.1) | 38% | 10% | 28% | 14% | 14% | 17% |
| Clustering coefficient (CC) |  |  |  |  |  |  |  |
|  | PWIAAFT (0) | 40% | 18% | 31% | 3% | 34% | 38% |
|  | PWIAAFT (0.03) | 45% | 13% | 31% | 7% | 34% | 31% |
|  | PWIAAFT (0.1) | 40% | 20% | 24% | 7% | 31% | 24% |
| Transitivity (TRANS) |  |  |  |  |  |  |  |
|  | PWIAAFT (0) | 45% | 18% | 38% | 7% | 34% | 34% |
|  | PWIAAFT (0.03) | 45% | 18% | 31% | 7% | 34% | 38% |
|  | PWIAAFT (0.1) | 40% | 23% | 34% | 10% | 34% | 31% |
